# The Effect of COVID‐19 Pandemic Restrictions on Laboratory Monitoring of Lithium Treated Outpatients in the Netherlands: A Controlled Interrupted Time Series Analysis

**DOI:** 10.1002/pds.70349

**Published:** 2026-03-12

**Authors:** Merel Tonn, Tim Bognàr, Ralph Kupka, Ingeborg Wilting, Mariette Nederlof, Toine Egberts, Arief Lalmohamed

**Affiliations:** ^1^ Department of Clinical Pharmacy University Medical Center Utrecht Utrecht the Netherlands; ^2^ Department of Clinical Pharmacy Amphia Hospital Breda the Netherlands; ^3^ Department of Psychiatry, Amsterdam Public Health Research Institute Amsterdam UMC, Vrije Universiteit Amsterdam the Netherlands; ^4^ National Health Care Institute Diemen the Netherlands; ^5^ Division of Pharmacoepidemiology and Clinical Pharmacology Utrecht Institute for Pharmaceutical Sciences, Utrecht University Utrecht the Netherlands

**Keywords:** Covid‐19, laboratory, lithium, monitoring, pandemic, time‐series

## Abstract

**Introduction:**

The COVID‐19 pandemic restricted healthcare access. Mental illness in combination with isolation and fear of the virus possibly decreased routine monitoring for lithium using patients during the lockdown. Our aim was to study if monitoring frequencies and serum level values for lithium, TSH (thyroid stimulating hormone), and renal function changed during the COVID‐19 pandemic.

**Methods:**

Using the PHARMO database (laboratory, pharmacy, and hospitalization data), we identified lithium users in The Netherlands over 2 years (14 October 2018–14 October 2020). The first year served as the control period; the second year was divided into pre‐COVID, lockdown, and post‐lockdown segments. A time series analysis with a linear regression model was performed to test for differences in monitoring frequency and aberrant serum levels at the beginning of the lockdown (immediate effect) and during the lockdown (post‐lockdown trend, effect over 12 weeks).

**Results:**

We identified 2835 patients using lithium. Monitoring measurements declined by 52% (7.74% vs. 4.01%) for lithium serum levels, 28% (5.56% vs. 4.20%) for TSH, and 26% (17.7% vs. 13.2%) for renal function in the first week of lockdown. These reductions were statistically significant compared to the control period. Monitoring for all measurements gradually recovered during lockdown. Changes in aberrant serum levels were not statistically different during the exposure and control periods.

**Conclusion:**

Monitoring for lithium, TSH and renal function declined at the beginning of lockdown. However, it is unlikely that postponed laboratory measurements had clinically relevant negative treatment effects since no differences in aberrant serum levels were identified.

## Key Points

- Monitoring measurements for lithium patients declined strongly in the first week of the COVID‐19 lockdown.
- The number of measurements declined with 52% for lithium serum levels, 28% for TSH, and 26% for renal function.
- No increase in sub‐ and supratherapeutic lithium serum levels, out of normal range TSH or eGFR below 60 mL/min was observed.
- Consequenses of reduced access to monitoring were limited on a population based‐level during the lockdown period.
- Guidance by (mental) health care professionals is important during periods of limited access to healthcare.

## Plain Language Summary

Lithium is a psychotropic drug which is used in a wide range of psychiatric conditions. Additional monitoring measurements for drug serum levels, thyroid function and renal function are required for patients who use lithium to ensure effectiveness and prevent side effects or toxicity. During the COVID‐19 lockdown period, access to healthcare was restricted. This research showed that monitoring measurements during the lockdown period declined. Despite a decline in measurements, there was no increase in measurements that were out of normal range during and after the lockdown period. Therefore, the consequences of reduced access to laboratory monitoring during a relatively short time period had limited impact on a population‐based level.

## 1 Introduction

Lithium is a psychotropic drug which is frequently used in a wide range of psychiatric conditions. Particularly as long‐term preventive maintenance treatment in bipolar mood disorder [1, 2]. The drug should however be monitored carefully, because of its narrow therapeutic range and its wide intra‐ and interpatient variability [3, 4]. During the COVID‐19 pandemic, access to healthcare was restricted since lockdown restrictions limited patient's mobility [5]. Healthcare systems were challenged and continuity of visits to health facilities such as doctors' appointments, laboratory, and prescription refills were compromised. Combined with fear of being exposed to the virus, healthcare access was reduced [6, 7, 8, 9]. Furthermore, patients with pre‐existing (severe) mental illness were vulnerable to relapse during the pandemic due to high susceptibility to stress and loneliness associated with COVID‐19 restrictions (e.g., social distancing and home isolation) [10, 11, 12, 13]. A study on healthcare access during the COVID‐19 pandemic in Japan found a decrease in hospital visits of approximately 10%–20% for psychiatric outpatients [14]. This increases risk of treatment non‐compliance and disrupted lithium use patterns and monitoring [12]. Previous research has shown that, before COVID‐19, in 50%–70% percent of lithium outpatients some form of laboratory monitoring was done where lithium serum levels, thyroid stimulating hormone (TSH) or creatinine were monitored in a 6 month period [15]. This number was lower when looking at full laboratory monitoring for lithium, renal function and thyroid function altogether (i.e., as recommended by national guidelines) [15, 16]. As a consequence of the restrictions during the pandemic, compliance to lithium monitoring could have decreased, and it is currently unknown if the pandemic has affected lithium monitoring. Therefore, this study aimed to evaluate how COVID‐19 pandemic restrictions influenced laboratory monitoring in outpatients treated with lithium, by describing trends over time and exploring potential differences between the control and exposure periods.

## 2 Methodology

### 2.1 Settings and Source Population

For this observational study in The Netherlands, we used the Dutch PHARMO Database Network to select all patients with a lithium prescription between 14 October 2018 and 14 October 2020. PHARMO is a population‐based network which uses different electronic data sources from primary and secondary healthcare settings in The Netherlands. For this study, the Out‐patient Pharmacy Database, Clinical Laboratory Database, and Hospitalization database were used. The Out‐patient Pharmacy Database comprises prescribed healthcare products dispensed by out‐patient pharmacies. Information on product type, date, strength, dosage regimen, quantity, and prescriber were extracted from this database. Laboratory findings, including creatinine, estimated glomerular filtration rate (eGFR), TSH, and lithium serum levels, were extracted from the Laboratory Database. Furthermore, the Hospitalization database includes data on admission and discharge for every patient. This was used to determine periods of hospitalization.

### 2.2 Study Population and Exposure

**FIGURE 1:**
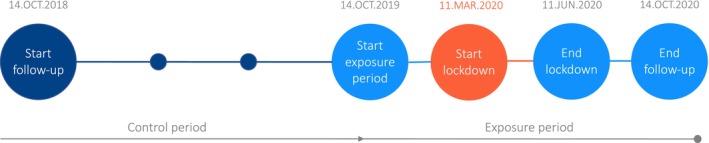
Schematic overview of the study period.

Patients with at least one lithium dispensing (ATC code N05AN01) between 14 October 2018 and 14 October 2020 were included in the study. Patients had to be present in the database at least 1 year prior to the first lithium dispensing in order to be able to distinguish between incident and prevalent users of lithium. Patients were included if their records were available in all three databases throughout the entire study period. Based on the periods of interest, the following patients were selected from the PHARMO database:

- Exposure period: patients with at least one lithium dispensing before, during or after the onset of the COVID‐19 measures in the Netherlands from 14 October 2019 till 14 October 2020. Within this period, the following three time‐intervals of interest were defined (Figure 1):
  ○ Pre‐COVID‐19: 14 October 2019 to 11 March 2020
  ○ COVID‐19 lockdown: 12 March 2020 to 01 June 2020 (active phase of Dutch COVID‐19 lockdown measures).
  ○ Post‐lockdown: 02 June 2020 to 14 October 2020 (COVID‐19 related measures were eased)
- Control period: patients with at least one lithium dispensing in the control period from 14 October 2018 to 14 October 2019 (1 year prior to the pandemic). Similar time intervals were defined to compare the control and exposure period.

The analysis was based on a single time series, comparing the pre‐lockdown period (referred to as the control period) with the lockdown period (exposure period). No separate control group was included; the control period represents the same population prior to the intervention. If patients were present in the Out‐patient Pharmacy database, but not in the Laboratory or Hospital Database, their records were excluded.

### 2.3 Definitions and Endpoints

A total of 6 parameters were determined for each individual patient. These parameters were calculated weekly, with each data point representing a one‐week period. Monitoring outcomes were based on national guidelines [17, 18] and included:

- Monitoring measurements, defined as the number of unique measurements per 100 current users per week, for:
  ○ Lithium
  ○ TSH
  ○ eGFR/creatinine
- Serum levels within the (therapeutic) range:
  ○ Lithium: the percentage of patients with lithium serum levels within the therapeutic range of 0.40–1.2 mmol/L, according to the Dutch guideline for bipolar disorders [18]. Therapeutic serum levels were calculated per number of lithium measurements per week.
  ○ TSH: the percentage TSH levels in the normal range per number of total TSH measurements. The normal range for TSH serum levels, as per Dutch general practitioner guidelines for thyroid disorders, is between 0.35 mUI/L and 4.0 mUI/L. [19].
  ○ eGFR: the number of eGFR measurements (CKD‐EPI, MDRD, and Cockroft Gault) above 60 mL/min(/1.73 m^2^) per total number of eGFR measurements.

Treatment episodes were calculated for each patient using the R‐package AdhereR (version 0.8.1) [20]. If the difference between theoretical end date and subsequent dispensing was longer than 30 days, the date of subsequent dispensing was considered as the start of a new treatment episode. Furthermore, overlap in treatment episodes within the same patient was considered by adding the number of overlapping days to the end of the treatment episode. The number of current lithium users was calculated for each week of the time series by calculating the number of patients that had a treatment episode per week.

### 2.4 Statistical Analysis

The statistical analysis was performed in R‐studio (version 2022.02.0, R version 4.1.2 (2021‐11‐01)). Descriptive statistics were used to provide an overview of baseline characteristics for all outpatients treated with lithium, stratified by calendar time. Frequencies and proportions were calculated for categorical variables and means (standard deviations) were provided for normally distributed continuous data. Differences between normally distributed continuous variables were tested using a *T*‐test. Differences between non‐normally distributed continuous variables were tested using the Wilcoxon signed rank test. A *χ* ^2^ test was used for differences between categorical variables. To assess the impact of COVID‐19 pandemic lockdown measures, a controlled interrupted time series analysis was performed [21, 22, 23]. For both the control period (pre‐COVID‐19) and the COVID‐19 period, data points on all outcomes were calculated weekly (averaging step).

A parallel control period was included, starting on the same calendar day as the exposure period, to account for seasonal effects, underlying trends, and time‐varying factors by enabling direct comparison (e.g., February 1st 2019 versus February 1st 2020).

**FIGURE 2:**
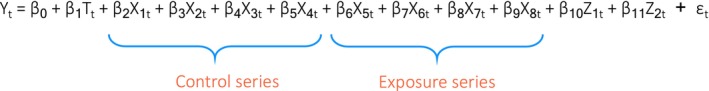
The linear regression model that describes the time series with parameters for the control and exposure period. *T* = continuous variable for time, *X*_1_ and *X*_5_ = binary variable for lockdown/immediate effect (yes/no). *X*_2_ and *X*_6_ = continuous variable for time after the lockdown/post‐lockdown trend (0 before lockdown). *X*_3_ and *X*_7_ = binary variable for the lift of the lockdown/immediate effect (yes/no). *X*_4_ and *X*_8_ = continuous variable for time after the lift of the lockdown/post‐lockdown trend (0 before lift). *Z*_1_ = number of patients admitted to the hospital per week. *Z*_2_ is an indicator for control or exposure group. The term ε describes random error.

Subsequently, linear regression was used for segmented regression analysis on all outcome variables in both the exposed and control group. Figures 2 and S1 provide a schematic overview of the controlled interrupted time series analysis. The statistical model that was used is described with the following formula:

In this formula, *Y* is the outcome, *T* a continuous variable that indicates time, *X*_1_ and *X*_5_ are binary variables which indicate if the observation was collected before or after the lockdown, and *X*_2_ and *X*_6_ are continuous variable that shows the time that has passed since the lockdown, for the control and exposure group respectively. Similarly, *X*_3_ and *X*_7_ are binary variables that indicate the moment the lockdown was lifted. *X*_4_ and *X*_8_ show the time that has passed since the lockdown was lifted. *Z*_1_ is a binary variable which indicates the period (control/exposure) and allows the exposure period to have a different intercept. To adjust for varying numbers of (general) hospital admissions (see potential confounders below) we incorporated this in the model as variable *Z*_2_. The term ε describes random error. As described, the time series analysis was performed in a single model. However, for graphical representation, we chose to overlay the exposure period on the control period in the figures to clearly illustrate the differences. Finally, we determined if there was an immediate effect and/or a post‐lockdown trend (i.e., effect seen during the 12 weeks lockdown period). Therefore, the linearHypothesis () test from the “car” package in R was performed on the estimates for “lockdown” (immediate effect) and “time since lockdown” (post‐lockdown trend) to test for differences between the control and exposure period (*β*_2_ *X*_1t_ versus *Β*_6_ *X*_5t_ and *β*_3_ *X*_2t_ versus *β*_7_ *X*_6t_ etc.) [24]. To provide more insight in differences between lab measurements, we conducted a before‐after comparison which can be found in Table S1 and Figure S3. The Wilcoxon signed rank test was used to test for differences in laboratory measurements.

### 2.5 Potential Confounders and/or Effect Modifiers

To control for bias, hospital admissions were taken into account in the analysis. Laboratory data of patients who were admitted to the hospital were not available in the PHARMO laboratory database during admission. As this could introduce bias, hospital admissions were added as a variable to the regression model. Furthermore, we performed a subgroup analysis for different age groups to identify possible effect modification.

### 2.6 Subgroup Analysis

Older patients were more vulnerable to a COVID‐19 infection, and therefore the immediate and sustained lockdown effect might differ per age group [25, 26]. To study potential age effects, we performed a subgroup analysis for three age groups (< 50 year, 50–70 years, and > 70 years).

### 2.7 Sensitivity Analysis

To assess the robustness of our findings, we conducted a sensitivity analysis in which the variable for hospitalization was excluded from the models. The results of this analysis are provided in the Supporting Information.

### 2.8 Ethics Statement

All methods were carried out in accordance with relevant regulation and guidelines. Because the study was non‐experimental, written informed consent was not required. The study was approved by the local medical ethics committee under reference number 22/586 on April 15 2022.

## 3 Results

**FIGURE 3:**
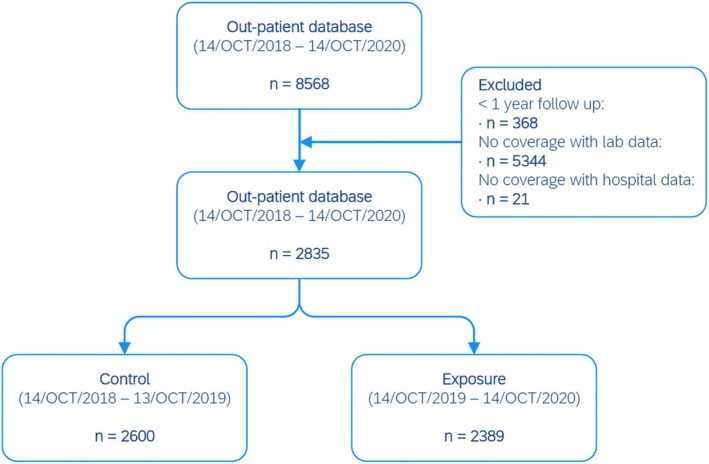
Flowchart of included patients.

From the outpatient database, 8568 unique patients using lithium were identified. Of these lithium users, 2835 individuals were mutually present in all databases and had a follow up of ≥ 1 year. Finally, 2600 users were included into the control group, and 2389 users into the exposure group (Figure 3).

### 3.1 Baseline Characteristics

Patient characteristics were equally distributed across both groups (Table 1). Most patients were female (59%), with a median age of 59 years. Approximately 10% of the patients in each group were admitted to the hospital at least once. Median duration of hospital admission was 1 day in both groups. During the study period, the number of current lithium users declined by 32% between start and end of follow up, which is caused by a decline in database coverage rather than a decrease in lithium use (Figure S2).

**TABLE 1:** Baseline characteristics.

	Control period (*n* = 2600)	Exposure period (*n* = 2389)	*p*
Period	14 Oct 2018–13 Oct 2019	14 Oct 2019–13 Oct 2020	
Age (median (IQR))	59 (50–70)	59 (49–69)	0.3070
Female (*n* (%))	1533 (59.0)	1413 (59.2)	0.8721
Admitted to hospital at least once (*n* (%))^a^	280 (10.8)	221 (9.3)	0.0827
Duration of admission (median (IQR))^b^	1 (0–5)	1 (0–5)	0.0541

^a^ Number of patients that were admitted at least one time in the period.

^b^ Duration of Hospital admission in days (24 h.).

### 3.2 Laboratory Monitoring

#### 3.2.1 Monitoring Measurements

**FIGURE 4:**
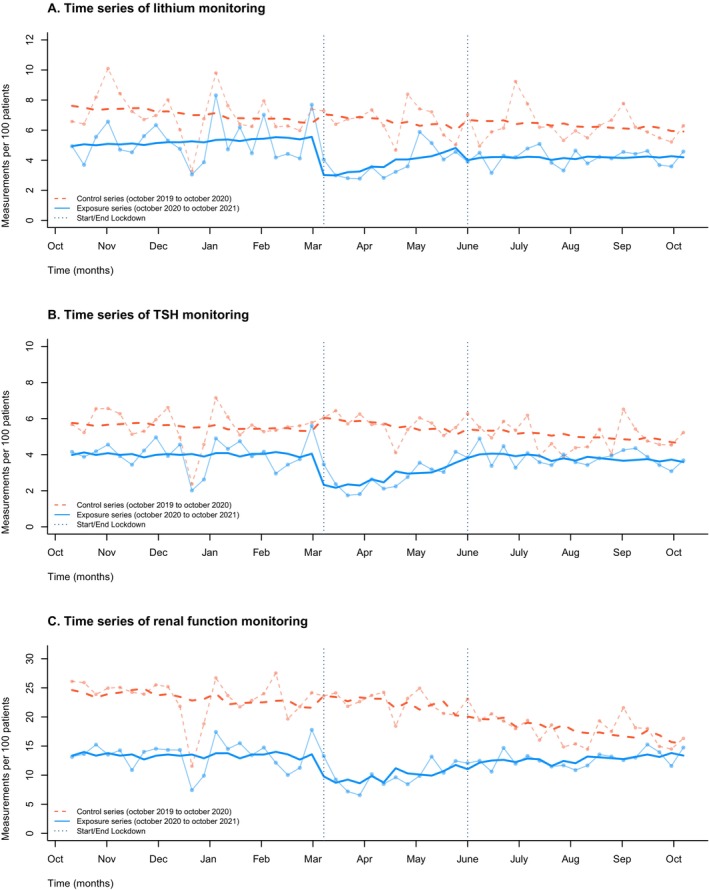
Time series analysis performed in a single model, with the exposure period (solid line, blue) overlaid on the control period dashed line, orange to visually emphasize differences between the two. The vertical dashed lines represent the beginning and the end of the lockdown period. Data points were calculated weekly. (A) Time series of lithium monitoring. (B) Time series of TSH monitoring. (C) Time series of renal function monitoring. TSH: Thyroid stimulating hormone. eGFR: Estimated glomerular filtration rate. Analysed as a single model, but presented as an overlay for graphical presentation.

For lithium, TSH and renal function, the weekly number of measurements per 100 users declined in the first week of the lockdown. The timeseries datapoints and fitted values of the linear regression model for all monitoring endpoints are shown in Figure 4 for the control and exposure series.

In the first lockdown week the number of measurements per active lithium users declined by 48% for lithium serum level measurements (from 7.64%, *n* = 147 to 4.01%, *n* = 77), by 24% for TSH (from 5.56%, *n* = 107 to 4.2%, *n* = 77), and by 25% for renal function (from 17.7%, *n* = 340 to 13.2%, *n* = 253). Table 2 shows the result of the fitted time series linear regression model. The observed effect for entering the lockdown was estimated at an absolute change of −2.51% (95% CI: −4.11 to 0.92), −2.01% (95% CI: −3.08 to −0.94), and −4.73% (95% CI: −8.03 to −1.42) for lithium, TSH and renal function monitoring respectively. All model coefficients for entering the lockdown were significantly different from the coefficients in the control series (*p*‐values were 0.0465 (lithium), 0.0002 (TSH) and 0.0167 (renal function)). During the lockdown, the number of measurements per 100 patients restored gradually. For TSH measurements, the sustained lockdown effect differed between the exposure period and the control period. For lithium and TSH the post‐lockdown trend for monitoring did not differ significantly.

**TABLE 2:** Estimated influence of all model parameters on monitoring.

		Immediate effect			Sustained effect		
		Coefficient	Confidence interval	*p*	Coefficient	Confidence interval	*p*
Lithium monitoring	Exposure	−2.51	−4.11; 0.92	0.04864*	0.19	−0.00; 0.39	0.3796
	Control	−0.33	−2.04; 1.38		0.05	−0.20; 0.31	
Adequate lithium levels	Exposure	−5.74	−11.99; 0.51	0.08082	0.63	−0.15; 1.41	0.2704
	Control	1.81	−4.88; 8.51		−0.05	−1.04; 0.95	
TSH monitoring	Exposure	−2.01	−3.08; −0.94	0.000247	0.14	0.01; 0.27	0.04411*
	Control	0.70	−0.45; 1.84		−0.07	−0.24; 0.10	
Adequate TSH levels	Exposure	−8.20	−14.46; −1.95	0.1182	1.22	0.43; 2.00	0.6397
	Control	−1.43	−8.14; 5.27		0.93	−0.07; 1.92	
Renal function monitoring	Exposure	−4.73	−8.03; −1.42	0.01749*	0.34	−0.07; 0.76	0.2527
	Control	0.71	−2.83; 4.26		−0.03	−0.56; 0.50	
Adequate eGFR levels	Exposure	3.79	−3.93; 11.52	0.7992	−0.10	−1.07; 0.86	0.4773
	Control	2.43	−5.85; 10.71		0.44	−0.79; 1.67	

Abbreviations: eGFR: estimated glomerular filtration rate; TSH: thyroid stimulating hormone.

** *p* < 0.001.

* *p* < 0.05.

#### 3.2.2 Serum Levels

**FIGURE 5:**
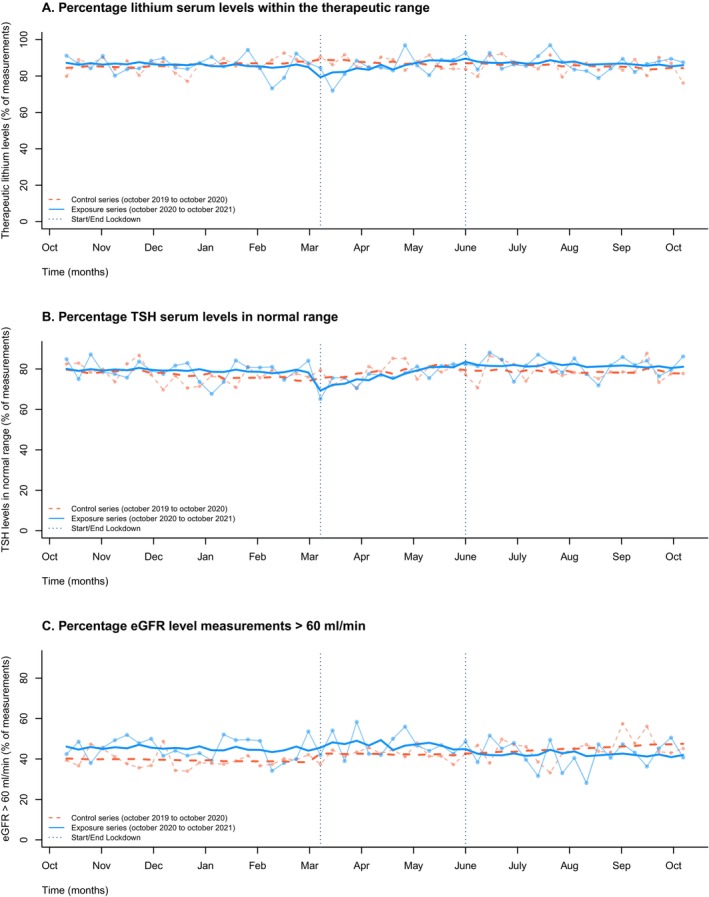
Time series of therapeutic/normal serum levels for lithium (A), TSH (B), and eGFR levels > 60 mL/min (C). Weekly data points are displayed, with the single statistical model represented by an overlay: The exposure period shown as a solid blue line and the control period as a dashed orange line. TSH: Thyroid stimulating hormone. eGFR: Estimated glomerular filtration rate.

We did not find a change in levels of lithium, TSH and eGFR when comparing levels at the beginning of the lockdown with the time period during the lockdown. Datapoints and fitted regression models for therapeutic lithium serum levels, normal TSH serum levels and eGFR > 60 mL/min are shown in Figure 5. Moreover, when looking at all measurements in the 12 weeks of lockdown, no differences sub‐ and supratherapeutic (lithium), out of normal range (TSH) or below 60 mL/min (eGFR) were observed (Table S1 and Figure S3).

### 3.3 Subgroup Analysis

Points estimates of immediate and post‐lockdown trends of the lockdown on monitoring were similar across all age groups (< 50, 50–70 and > 70 years). Results can be found in Tables S3 and S4, and Figures S4–S9. The effect was not statistically significant for all groups which may reflect a lack of statistical power due to a low number of measurements in the age groups < 50 and > 70 years.

## 4 Discussion

In the first week of the COVID‐19 lockdown, monitoring of lithium patients strongly declined. For lithium serum levels, the number of measurements declined by 48%. Monitoring of TSH and renal function declined by 28% and 26%, respectively. However, we did not find a substantial increase in serum levels outside the reference range.

These results are in line with various studies investigating laboratory monitoring of out‐patients during the COVID‐19 pandemic. First, Durant et al. found a decrease in total laboratory testing during the lockdown period, although test volumes for COVID‐19 diagnosis (e.g., PCR testing) and COVID‐19 management had increased. Test frequencies were measured weekly for all laboratory tests that were performed in their urban tertiary medical care center (United States) among all patients [27]. This observed immediate lockdown effect on monitoring of Durant et al. is similar to the effect we found for TSH and renal function. Second, Duce et al. reported that the interval between TSH monitoring tests was longer during the pandemic for patients using lithium, increasing from 17 weeks (median) to 21 weeks (median). They hypothesize this may also be the case for lithium serum level monitoring potentially resulting in increased psychiatric hospital admissions [28]. Furthermore, Rojas‐Velasquez et al. described two cases of supratherapeutic lithium levels during COVID‐19 infection [29]. In our study, we did not find an indication for increased numbers of supratherapeutic levels or increased number of hospital admissions due to extended monitoring intervals, nor did we find an increase in lithium serum levels out of therapeutic range. Third, Singh et al. studied changes in test volumes and laboratory utilization in inpatients and outpatients during the COVID‐19 pandemic using a time series analysis. They found three different patterns for the effect of the lockdown restrictions on laboratory monitoring among different diagnostic‐, screening‐ and monitoring tests [30]. The first pattern (for estradiol) reflects the trend which was found in our current study: a decrease at the beginning of the lockdown, followed by recovery of test volumes. The extent of decline in test volumes was also comparable between the results of our study and theirs. The second pattern, no change, (for neonatal bilirubin and group B Streptococcus), can be attributed to continuous birth counts, which is not influenced by the lockdown measures during the pandemic. The third pattern they identified (for HbA1c, whole blood lead level and Papanicolaou (Pap) smear) was an initial drop with only partial recovery of test volumes, which could indicate sustained suboptimal monitoring [30]. This partial recovery of test volumes is not in line with our results, as we observed a gradual (but complete) recovery in the volume of all three measurements. This contrasts with the consequences of the lockdown for cancer care. Fewer patients were seeking primary care leading to fewer cancer diagnoses, cancer treatments, and possibly to an increased number of cancer deaths [31, 32]. These studies showed that recovery for cancer care is in progress, but has not yet returned to pre‐COVID‐19 levels at the same rate as lithium monitoring in the current study [31].

Our time series analysis had several strengths. To our knowledge, this is the first large population‐based study to study the effect of the COVID‐19 pandemic on laboratory monitoring. Furthermore, the use of a large database with representative coverage provided sufficient and accurate data to perform a time series analysis. Because we had data on laboratory monitoring, lithium use and hospitalization, we could adjust for possible confounding resulting from hospitalization in the analysis. The laboratory database also included the actual lithium serum level measurements. Therefore, we could determine if lithium levels were within or outside the therapeutic range, allowing us to consider clinical impact. In addition to the identification of a different trend during the COVID‐19 lockdown, our data allowed us to demonstrate a statistically significant change in testing volumes compared to the previous year (control period). Several limitations also need to be considered. First, the coverage of the database decreased during the study period. This decrease was reflected by the number of lithium users, which declined by approximately one third (Figure S2). Therefore, we cannot rule out any bias that may have been introduced by the decrease in database coverage. For this reason, we calculated all endpoints relative to the number of current users. Second, we could not identify acute renal insufficiency because our laboratory database may not include all subsequent measurements for renal function (e.g., measurements during hospitalization). To estimate the consequences of COVID‐19 restrictions on renal function, we used a cutoff value for eGFR measurements, assuming that the number of patients with sufficient renal function in the population should remain constant over time if the pandemic had not occurred. Third, we did not have access to laboratory measurements for patients who were admitted to the hospital during hospitalization because hospital databases were not linked to the PHARMO database. In theory, a decrease in laboratory measurements could have been the consequence of an increase in hospital admissions. Accordingly, we included hospital admissions as a variable in the linear regression time series model to adjust for this effect.

Our findings may suggest that the delay in monitoring of lithium patients during the COVID‐19 pandemic period did not result in dysregulated serum levels for lithium and TSH and renal function loss. However, these results need to be interpreted cautiously because during the COVID pandemic the number of measurements also decreased. Therefore, dysregulated serum levels may have been unnoticed during that time. A retrospective single center study among patients using warfarin in a Turkish population showed that patients spent less time in therapeutic range during COVID‐19 compared to the time period before COVID‐19. In this study, 41% of outpatients lacked INR control during COVID‐19 [33].

Although we cannot draw conclusions about serum levels that were not measured in the current study, serum levels were not more frequently outside the therapeutic range when the number of measurements per 100 patients was restored. Furthermore, we suspect that patients who were vulnerable to relapse or were susceptible to fluctuating serum levels were more likely to be monitored during the lockdown period. For all outcomes, monitoring had (almost) returned to pre‐lockdown levels without an increase in aberrant serum levels. This suggests that the Dutch monitoring system was resilient and lithium users were monitored sufficiently during the lockdown.

Although we suspect that postponement of laboratory measurements had limited influence on serum levels, lithium users might still have experienced reduced care during the lockdown. Our results do not include clinical outcomes such as occurrence of relapses of depressive or manic episodes. A study of healthcare access in Japan during the COVID‐19 pandemic reported an excess in hospital admissions in psychiatric care in April and May 2020 [14]. This indicates that there has been a possible deterioration in mental health due to the pandemic. Hernández‐Gómez et al. have suggested recommendations for mental healthcare professionals who provide treatment for patients with bipolar disorder in times of health crisis. These recommendations include reviewing adherence to medical treatment during isolation and education of patients on how to identify relapses among others [11]. The United Kingdom National Health Service (NHS) developed an information memo for lithium monitoring during COVID‐19 in which they recommend continuation of monitoring according to guidelines for patients who have been taking lithium for less than 12 months. According to the NHS, the monitoring interval of stable patients without COVID‐19 symptoms, who are on lithium treatment for over 1 year could be extended, but this has to be decided on a case by case basis [34]. Furthermore, Chandran et al. studied adherence to osteoporosis therapy during COVID‐19 and showed that the odds for being adherent during the lockdown were lower than pre‐COVID‐19. Moreover, patients who were monitored by an endocrinologist had higher odds of being adherent compared to other health care providers [35]. This effect might also apply to lithium users and stresses the importance of guidance during lithium treatment by (mental) health care professionals. In our study, reductions in laboratory monitoring had limited impact on monitored serum levels for lithium users. However, during periods of crisis and reduced healthcare, patients are presumably more vulnerable to relapses and should be guided by their health care professionals.

## 5 Conclusions

In the first week of COVID‐19 lockdown, measurements for lithium serum levels were halved, whereas TSH and renal function monitoring diminished by a third. Despite this decline in laboratory monitoring, the current study did not find a substantial increase in the frequency of serum levels outside the reference range. Therefore, consequences of reduced access to laboratory monitoring, during a relatively short time period, had a limited effect on measured serum levels at a population‐based level.

## Author Contributions

**Merel Tonn:** conceptualization, data curation, formal analysis, methodology, visualization, writing – original draft, writing – review and editing. **Tim Bognàr:** conceptualization, formal analysis, methodology, supervision, writing – review and editing. **Ralph Kupka:** conceptualization, methodology, writing – review and editing. **Ingeborg Wilting:** conceptualization, writing – review and editing. **Mariette Nederlof:** conceptualization, writing – review and editing. **Toine Egberts:** conceptualization, methodology, supervision, writing – review and editing. **Arief Lalmohamed:** conceptualization, data curation, methodology, supervision, formal analysis, writing – review and editing.

## Funding

The authors have nothing to report.

## Conflicts of Interest

The authors declare no conflicts of interest.

## Supporting information


**Data S1:** Supporting Information.

## Data Availability

Data are not publicly available, and access may be requested through www.pharmo.nl.
