# Supplementary material for: The Effect of COVID‐19 Pandemic Restrictions on Laboratory Monitoring of Lithium Treated Outpatients in the Netherlands: A Controlled Interrupted Time Series Analysis
Source: Pharmacoepidemiol Drug Saf. 2026 Mar 12;35(3):e70349. doi: 10.1002/pds.70349 (PMC12982162; doi:10.1002/pds.70349)
Supplement: Supplementary file 1 — Data S1: Supporting Information. [file PDS-35-e70349-s001.docx]

# Supplementary material

1. **Controlled interrupted time series analysis**

If an effect is present during the exposure period, but not during the control period, the evidence for a relationship between the lockdown and outcome is more substantiated. If there is an effect in both the exposure and control series however, the effect may be subscribed to other factors.


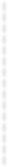

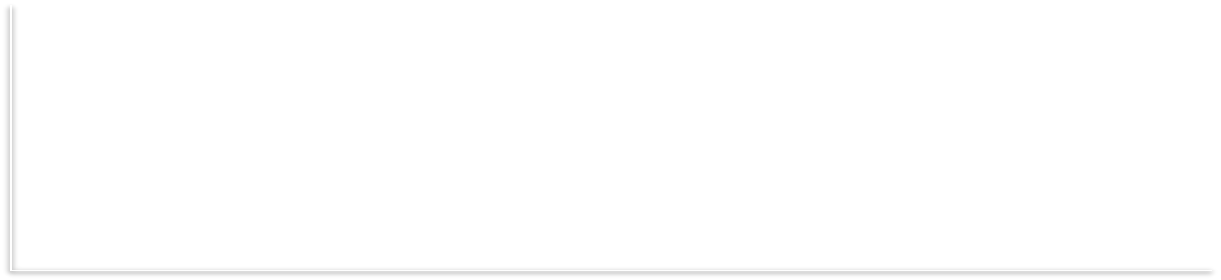

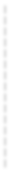

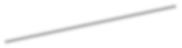

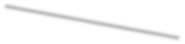

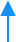

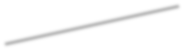

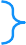


b

1 (slope)

b

5 (post-lockdown trend)

= difference between pre- and post slope

b

2 (immediate effect)

b

0 (intercept)

b

b

4 (immediate effect)

3 (post-lockdown trend)

= difference between pre- and post slope


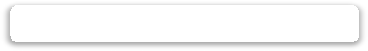

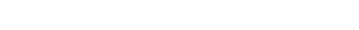


Period 1


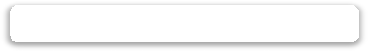

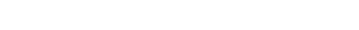


Period 2


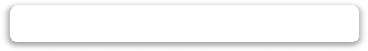

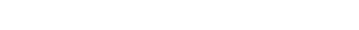


Period 3

Figure S1 - schematic overview of the interrupted time series analysis

# **Decrease in lithium users over time**


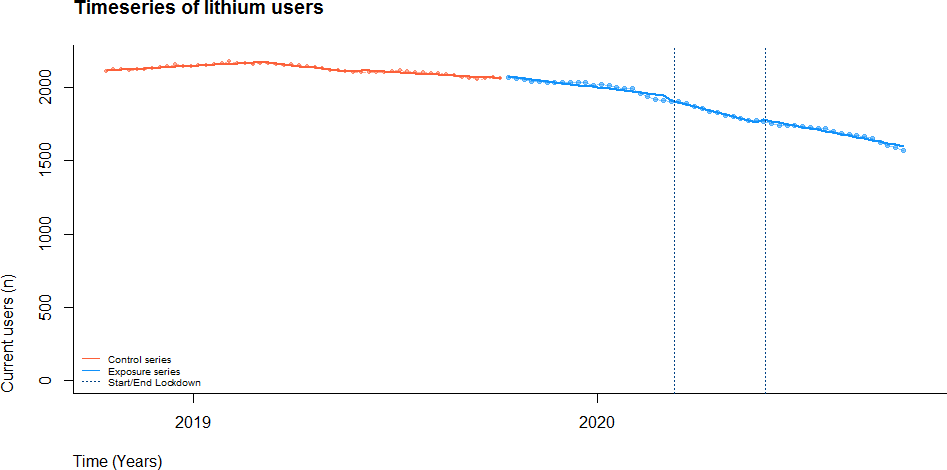


Figure S2 - Decrease in lithium users over time representing a decline in database coverage. Control period (orange line),

Exposure period (blue line).

# **Before- after comparison**

Comparison of the number of measurements per lithium user during 12 lockdown weeks (exposure period) to 12 weeks during the same time-period of the previous year (control period).

Table S1 - Medians of measurements that were out of therapeutic/normal ranges (%).

|  | Control | Exposure | P-value |
| --- | --- | --- | --- |
| Subtherapeutic lithium | 9.28 % | 9.95 % | 0.242 |
| Supratherapeutic lithium | 1.95 % | 2.20 % | 0.644 |
| Low TSH | 3.11 % | 3.85 % | 0.184 |
| High TSH | 16.79 % | 16.26 % | 0.887 |
| eGFR < 60 ml/min | 13.37 % | 15.55 % | 0.160 |

TSH: thyroid stimulating hormone. eGFR: estimated glomerular filtration rate.

1.
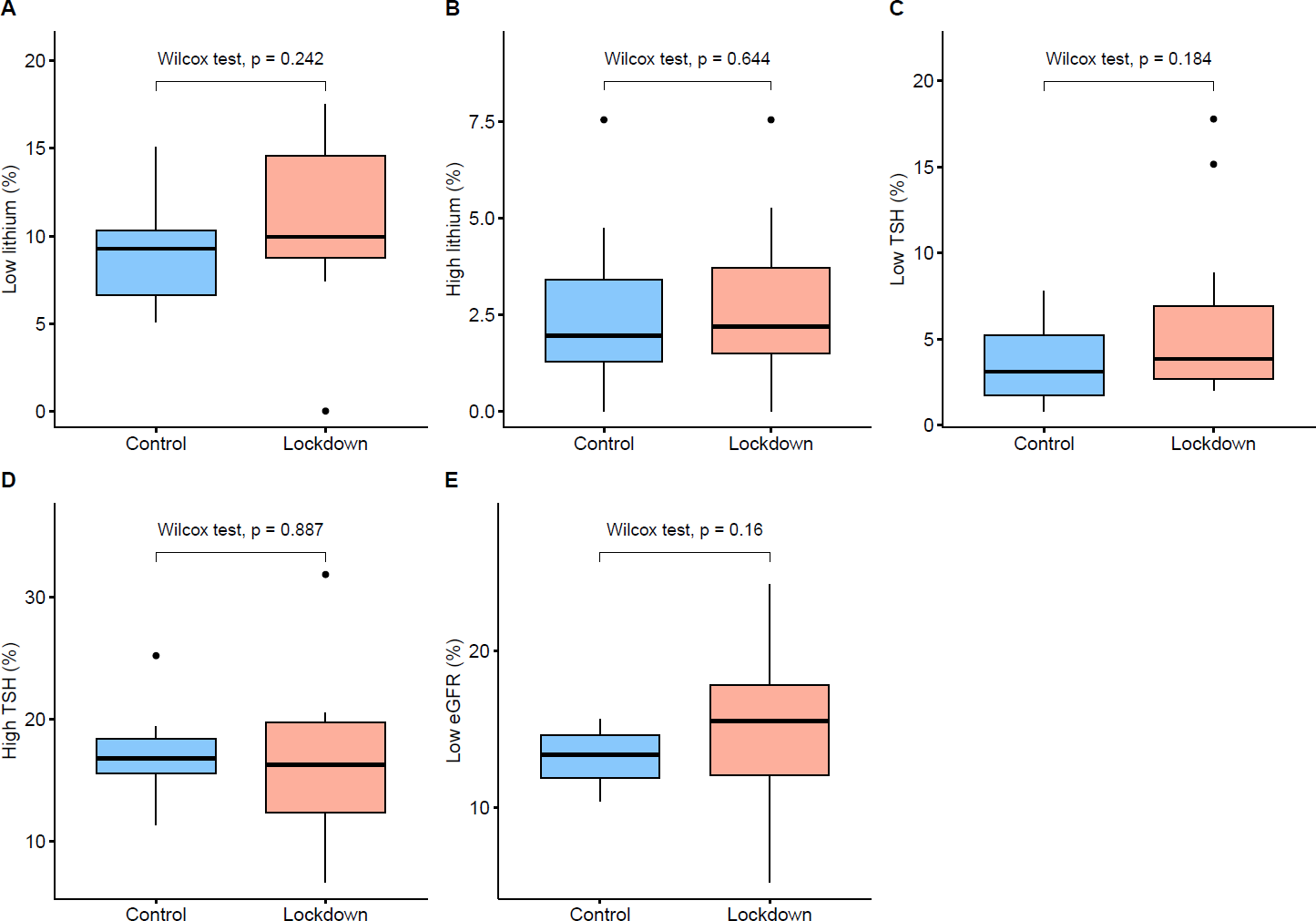
**Boxplots of differences**

*Figure S3 - boxplots of percentages of measurements out of therapeutic/normal range. A = low lithium serum levels. B = High lithium serum levels. C = Low TSH serum levels. D = High TSH serum levels. E = eGFR below 60 ml/min*

2

1. **Subgroup analysis**
   1. **Baseline characteristics of subgroups by age**

Table S2 - Baseline characteristics by age category.

|  | Control period | Exposure period |
| --- | --- | --- |
| Period | 14.OCT.2018 – 13.OCT.2019 | 14.OCT.2019 – 13.OCT.2020 |
| Subjects (n) |  |  |
| Age < 50 | 653 | 624 |
| Age 50 - <70 | 1308 | 1209 |
| Age >= 70 | 655 | 574 |
| Age (mean (SD)) |  |  |
| Age < 50 | 39.22 (7.83) | 38.94 (8.08) |
| Age 50 - <70 | 59.40 (5.51) | 59.43 (5.52) |
| Age >= 70 | 76.50 (5.37) | 76.32 (5.33) |
| Female (n (%)) |  |  |
| Age < 50 | 387 (59.3) | 380 (60.9) |
| Age 50 - <70 | 727 (55.6) | 672 (55.6) |
| Age >= 70 | 427 (65.2) | 370 (64.5) |
|  |  |  |
| *SD = Standard Deviation.* | | |

# **Model estimates**

Table S3 – Model estimates per age group.

| Age < 50 years | | Immediate effect | | Post-lockdown trend (12 weeks) | |
| --- | --- | --- | --- | --- | --- |
|  |  | Coefficient | P-Value | Coefficient | P-Value |
| Lithium monitoring rate | Exposure | -2.26 | 0.1709 | 1.47 | 0.7748 |
|  | Control | -0.76 |  | 0.93 |  |
| Adequate Lithium levels | Exposure | 4.40 | 0.5128 | -4.88 | 0.4412 |
|  | Control | -0.63 |  | 5.23 |  |
| TSH monitoring rate | Exposure | -2.83 | 0.007226 ** | 2.00 | 0.5030 |
|  | Control | 0.11 |  | 0.74 |  |
| Adequate TSH levels | Exposure | -2.69 | 0.9482 | 5.14 | 0.5567 |
|  | Control | -3.35 |  | 15.40 |  |
| Renal function monitoring rate | Exposure | -6.52 | 0.06529 | 4.17 | 0.8741 |
|  | Control | -0.83 |  | 5.00 |  |
| Adequate eGFR levels | Exposure | -7.23 | 0.9509 | 9.73 | 0.839 |
|  | Control | -7.82 |  | 13.09 |  |
| Age 50 - 70 years | | Immediate effect | | Post-lockdown trend (12 weeks) | |
|  |  | Coefficient | P-Value | Coefficient | P-Value |
| Lithium monitoring rate | Exposure | -2.66 | 0.02285 * | 2.50 | 0.2982 |
|  | Control | -0.38 |  | 0.73 |  |
| Adequate Lithium levels | Exposure | -9.32 | 0.06519 | 7.17 | 0.6051 |
|  | Control | 0.81 |  | 2.31 |  |
| TSH monitoring rate | Exposure | -1.91 | 0.0009684 *** | 1.80 | 0.05106 |
|  | Control | 0.73 |  | -0.86 |  |
| Adequate TSH levels | Exposure | -13.31 | 0.09484 | 17.07 | 0.7099 |
|  | Control | -1.75 |  | 12.67 |  |
| Renal function monitoring rate | Exposure | -4.37 | 0.04043 * | 3.67 | 0.5252 |
|  | Control | 0.66 |  | 1.00 |  |
| Adequate eGFR levels | Exposure | 2.54 | 0.9721 | -8.61 | 0.234 |
|  | Control | 2.27 |  | 6.72 |  |
| Age > 70 years | | Immediate effect | | Post-lockdown trend (12 weeks) | |
|  |  | Coefficient | P-Value | Coefficient | P-Value |
| Lithium monitoring rate | Exposure | -2.56 | 0.3254 | 1.74 | 0.6188 |
|  | Control | -0.05 |  | -0.44 |  |
| Adequate Lithium levels | Exposure | -6.15 | 0.1755 | 14.92 | 0.06198 |
|  | Control | 5.81 |  | -13.26 |  |
| TSH monitoring rate | Exposure | -1.17 | 0.07574 | 0.16 | 0.2038 |
|  | Control | 1.19 |  | -2.73 |  |
| Adequate TSH levels | Exposure | -0.40 | 0.9659 | 10.64 | 0.6367 |
|  | Control | -0.78 |  | 3.44 |  |
| Renal function monitoring rate | Exposure | -2.76 | 0.2423 | 0.67 | 0.1455 |
|  | Control | 2.26 |  | -10.03 |  |
| Adequate eGFR levels | Exposure | 4.82 | 0.8926 | 5.16 | 0.5321 |
|  | Control | 5.87 |  | -3.16 |  |

TSH: thyroid stimulating hormone. eGFR: estimated glomerular filtration rate.

# **Monitoring rates: age < 50**


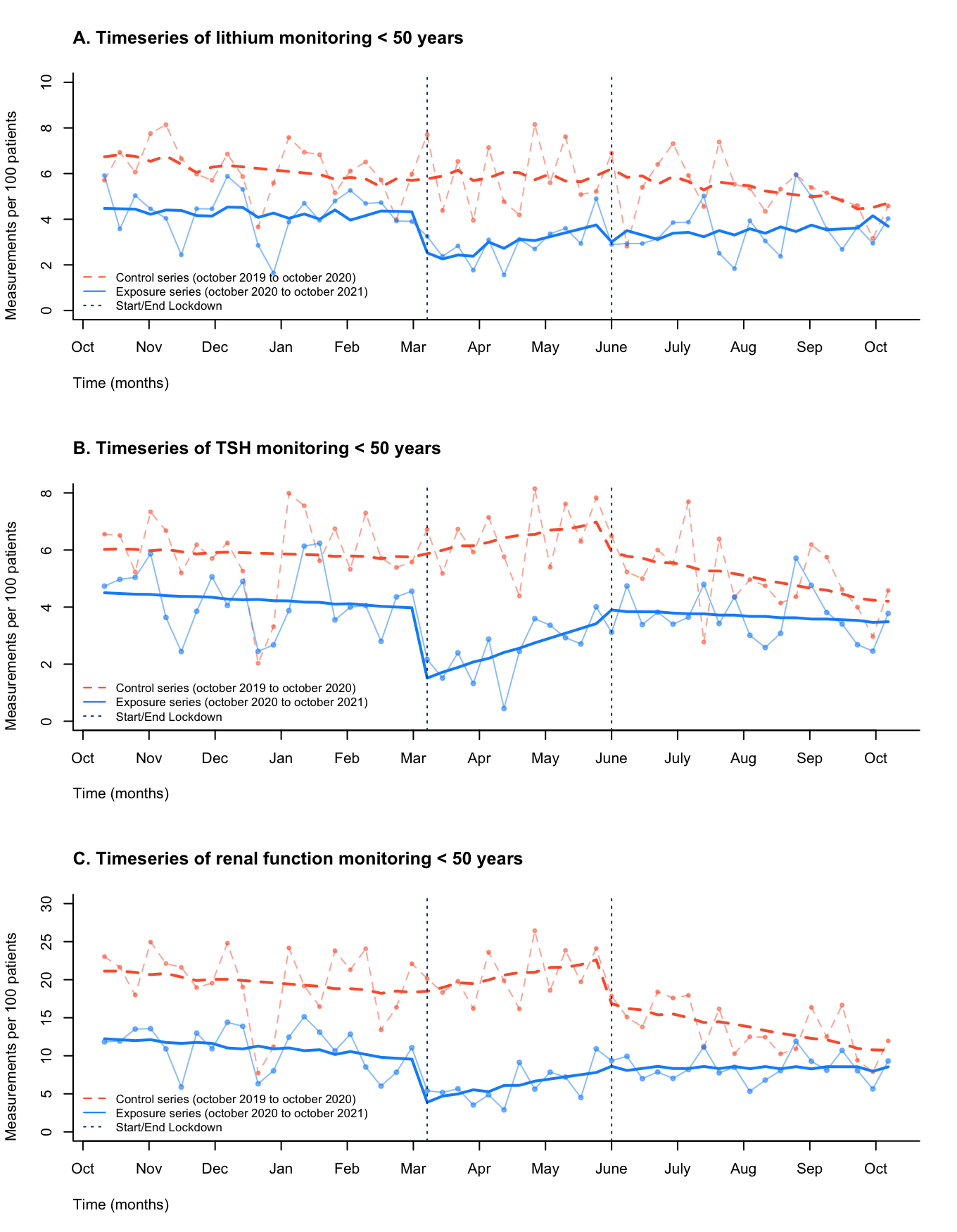


Figure S4 – Age < 50: Time series model of monitoring rate during the year of COVID-19, the exposure period (solid line, blue) and the control series (dashed line, orange). De vertical dashed lines represent the beginning and the end of the lockdown period. A: Time series of lithium monitoring rate. B. Time series of TSH monitoring rate. C. Time series of renal function monitoring rate. TSH: thyroid stimulating hormone. eGFR: estimated glomerular filtration rate.

# **Serum levels and eGFR age <50 year**


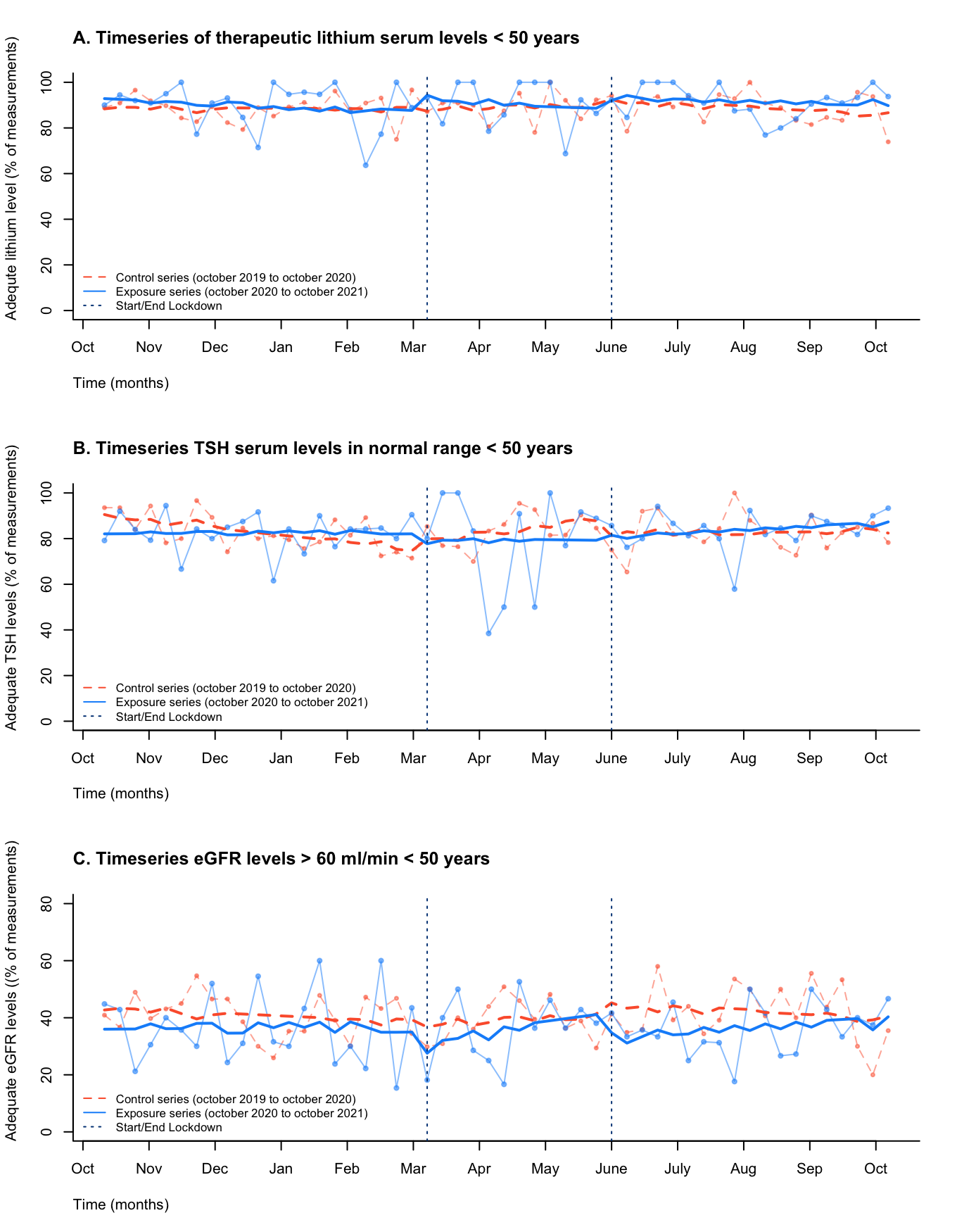


Figure S5 – Age < 50: Time series of therapeutic/normal (serum) levels for lithium (A), TSH (B), and eGFR levels > 60 ml/min (C). The data points are shown for every week with the statistical model as overlay for the exposure period (solid blue line) and control period (dashed orange line). TSH: thyroid stimulating hormone. eGFR: estimated glomerular filtration rate.

# **Monitoring rate age 50-70 year**


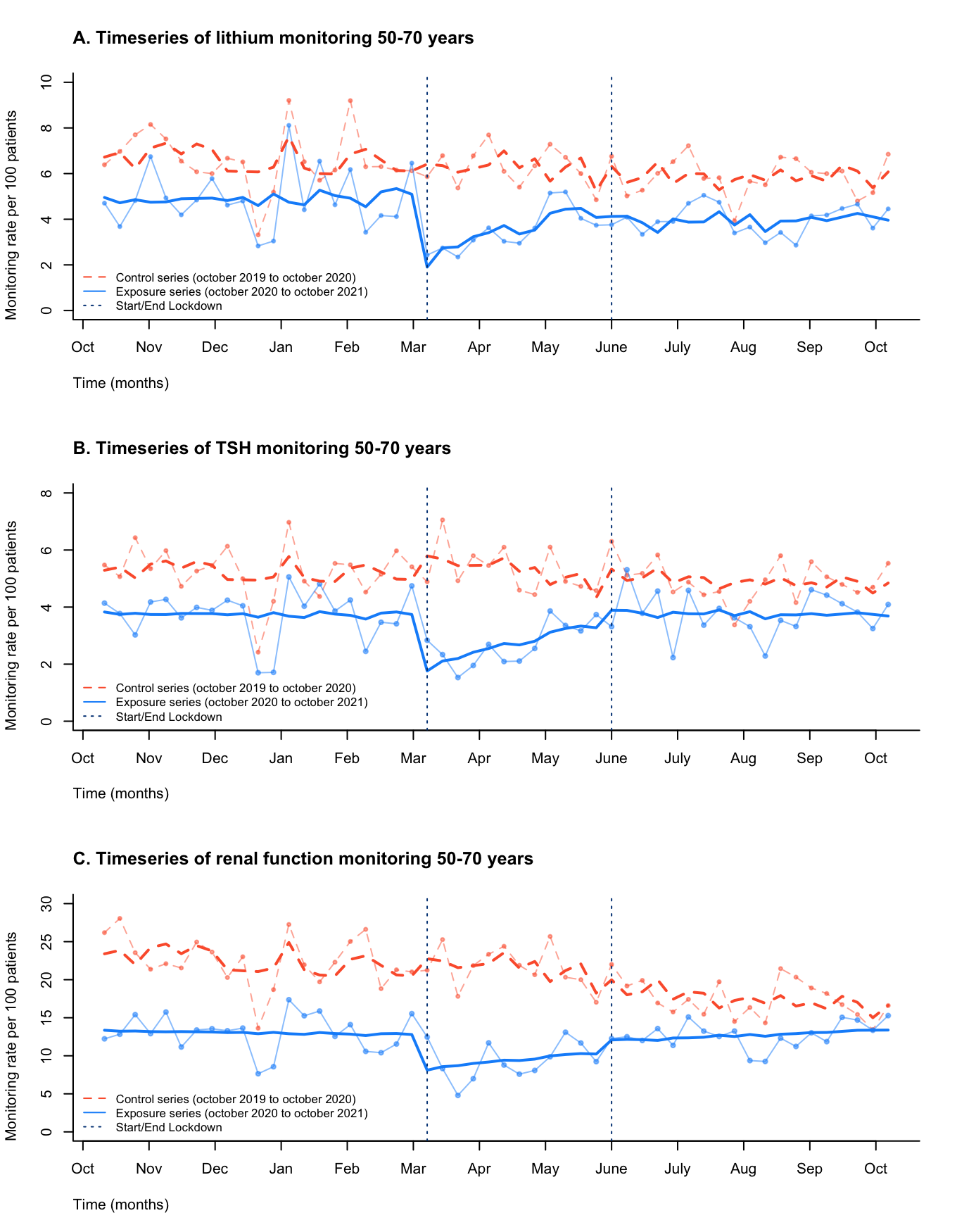


Figure S6 – Age 50-70: Time series model of monitoring rate during the year of COVID-19, the exposure period (solid line, blue) and the control series (dashed line, orange). De vertical dashed lines represent the beginning and the end of the lockdown period. A: Time series of lithium monitoring rate. B. Time series of TSH monitoring rate. C. Time series of renal function monitoring rate. TSH: thyroid stimulating hormone. eGFR: estimated glomerular filtration rate.

# **Serum levels and eGFR age 50-70 year**


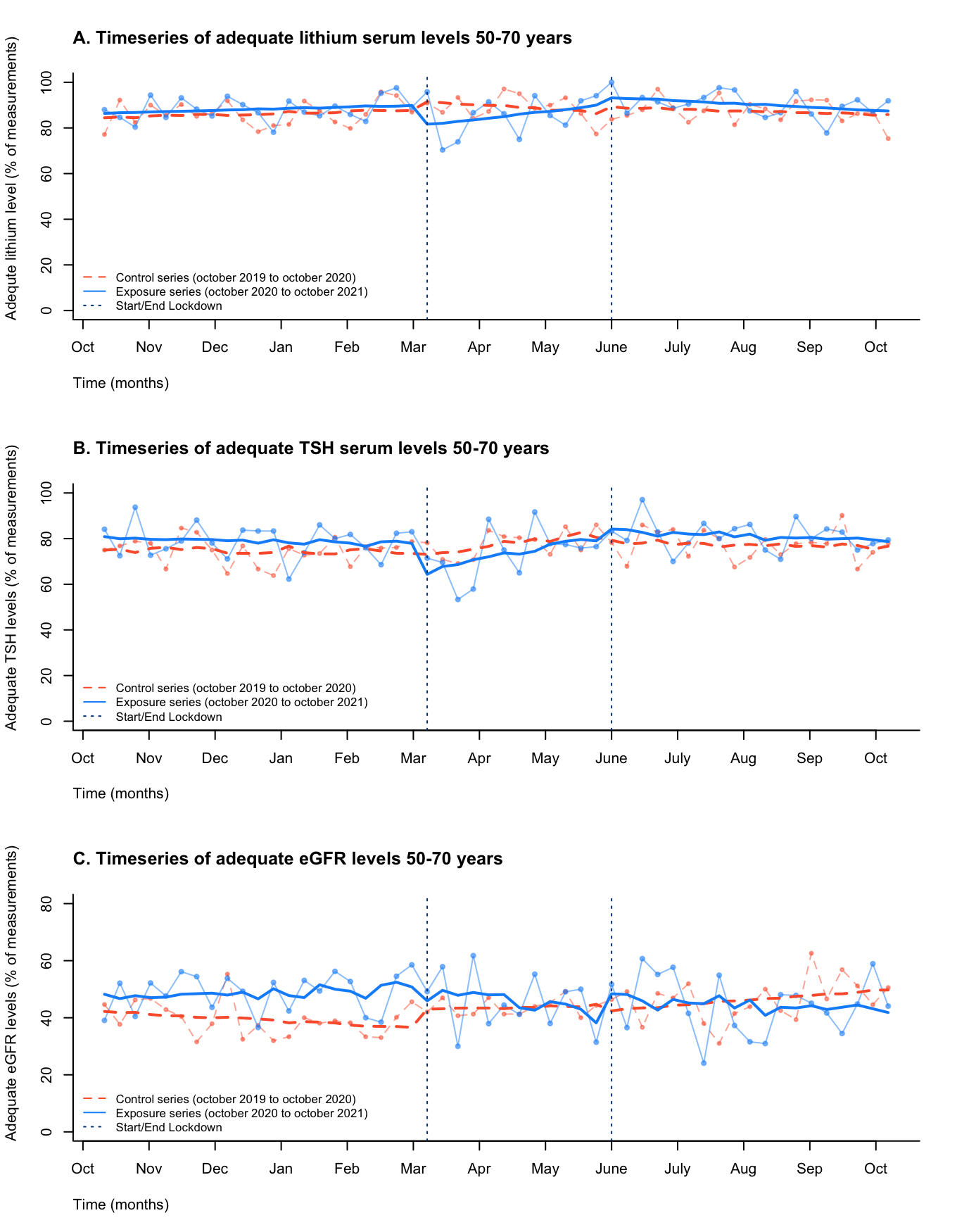


Figure S7 – Age 50-70: Time series of therapeutic/normal (serum) levels for lithium (A), TSH (B), and eGFR levels > 60 ml/min (C). The data points are shown for every week with the statistical model as overlay for the exposure period (solid blue line) and control period (dashed orange line). TSH: thyroid stimulating hormone. eGFR: estimated glomerular filtration rate.

# **Monitoring rate age >70**


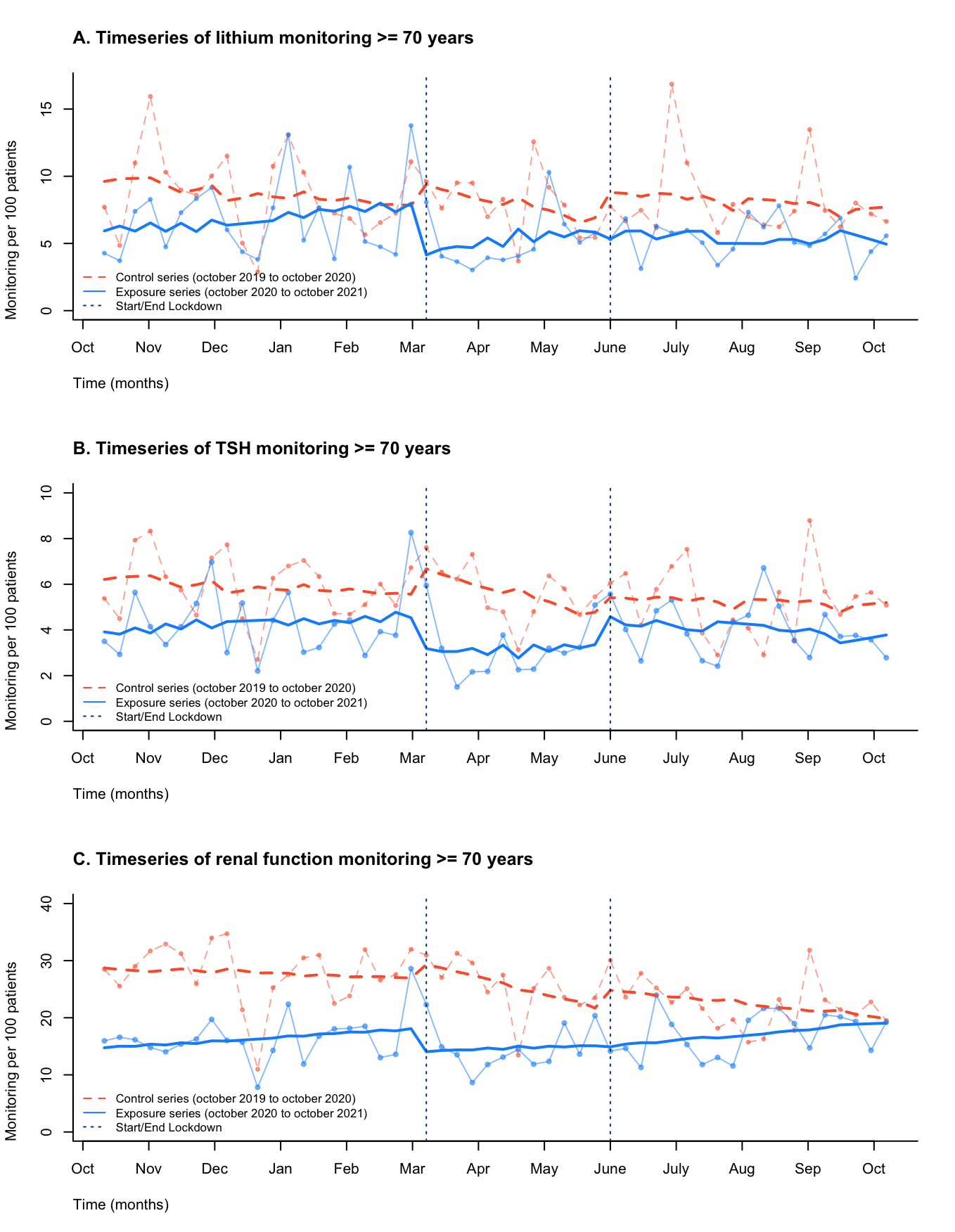


Figure S8 – Age > 70: Time series model of monitoring rate during the year of COVID-19, the exposure period (solid line, blue) and the control series (dashed line, orange). De vertical dashed lines represent the beginning and the end of the lockdown period. A: Time series of lithium monitoring rate. B. Time series of TSH monitoring rate. C. Time series of renal function monitoring rate. TSH: thyroid stimulating hormone. eGFR: estimated glomerular filtration rate.

# **Serum levels and eGFR age > 70**


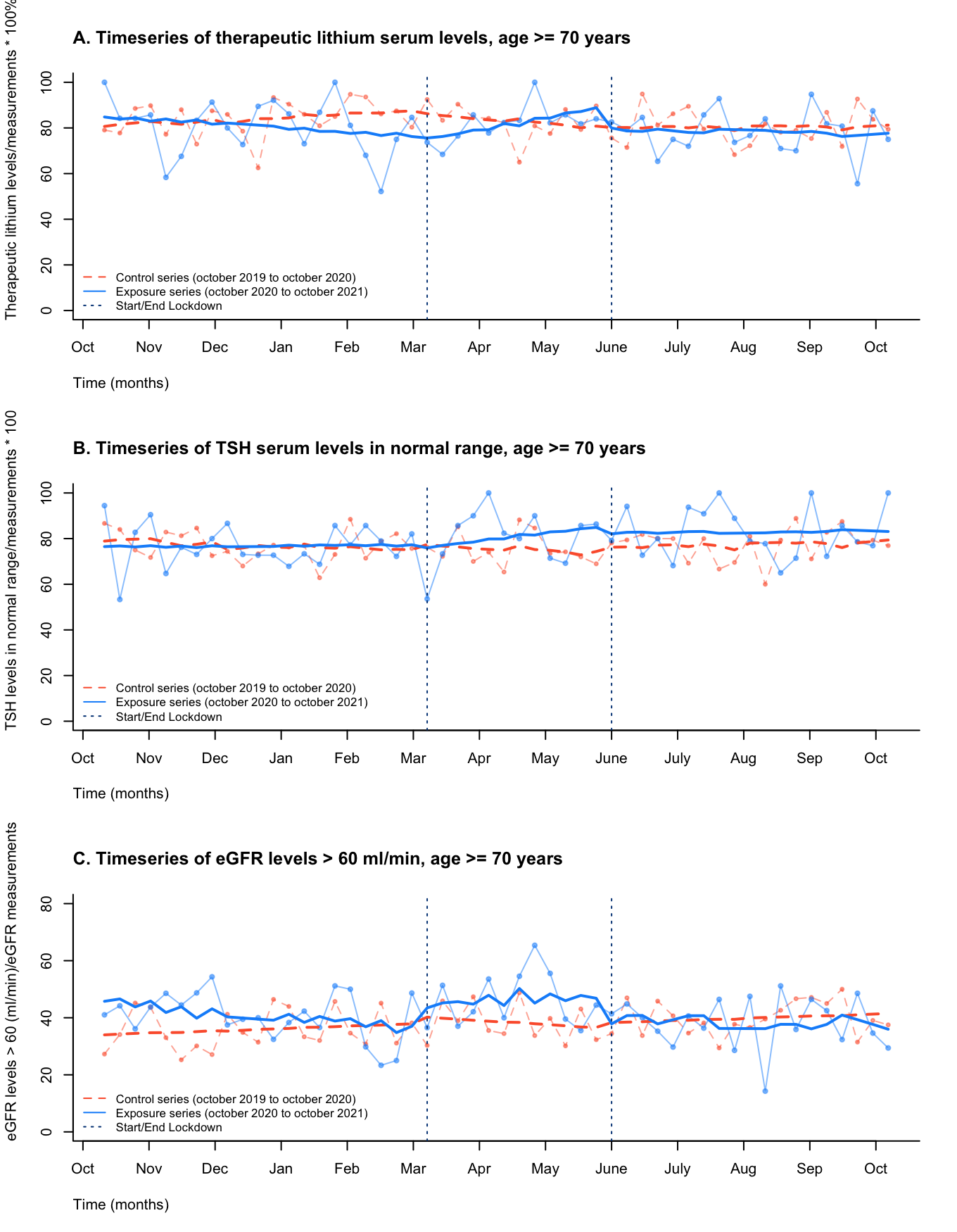


Figure S9 – Age > 70: Time series of therapeutic/normal (serum) levels for lithium (A), TSH (B), and eGFR levels > 60 ml/min (C). The data points are shown for every week with the statistical model as overlay for the exposure period (solid blue line) and control period (dashed orange line). TSH: thyroid stimulating hormone. eGFR: estimated glomerular filtration rate.

# **Decrease in monitoring rates during the first lockdown week for each parameter**

Table S4 – Decline in measurements in the first lockdown week, relative to the previous week.

| Subgroup | < 50 year | | | 50-70 year | | | > 70 year | | |
| --- | --- | --- | --- | --- | --- | --- | --- | --- | --- |
|  | Decline % | Before (n) | After (n) | Decline % | Before (n) | After (n) | Decline % | Before (n) | After (n) |
| Lithium | -16.67 | 18 | 15 | -62.50 | 64 | 24 | -41.54 | 65 | 38 |
| TSH | -52.38 | 21 | 10 | -40.43 | 47 | 28 | -28.21 | 39 | 28 |
| Renal  function | -50.98 | 51 | 25 | -20.13 | 154 | 123 | -22.22 | 135 | 105 |

TSH: thyroid stimulating hormone

1. **Sensitivity analysis (with and without adjustment for hospital admissions)**

Table 1 – Estimates adjusted and unadjusted for hospital admission for the immediate lockdown effect

|  | | **Immediate effect** | | | | | | | | | |
| --- | --- | --- | --- | --- | --- | --- | --- | --- | --- | --- | --- |
|  |  | **Adjusted** | | | | | **Unadjusted** | | | | |
|  |  | **Coefficient** | | **Confidence interval** | **P-value** | | **Coefficient** | | **Confidence interval** | | **P-value** |
| **Lithium monitoring** | **Exposure** | -2.51 | (-4.11 ; 0.92) | | | 0.04864 * | -2.41 | (-3.98 ; -0.84) | | 0.05799 . | |
|  | **Control** | -0.33 | (-2.04 ; 1.38) | | |  | -0.33 | (-2.04 ; 1.38) | |  |  |
| **Adequate Lithium levels** | **Exposure** | -5.74 | (-11.99 ; 0.51) | | | 0.08082 | -6.46 | (-12.66 ; -0.26) | | 0.05556 . | |
|  | **Control** | 1.81 | (-4.88 ; 8.51) | | |  | 1.81 | (-4.92 ; 8.54) | |  |  |
| **TSH monitoring** | **Exposure** | -2.01 | (-3.08 ; -0.94) | | | 0.0002474 *** | -1.91 | (-2.96 ; -0.85) | | 0.0003908 *** | |
|  | **Control** | 0.70 | (-0.45 ; 1.84_ | | |  | 0.70 | (-0.45 ; 1.84) | |  |  |
| **Adequate TSH levels** | **Exposure** | -8.20 | (-14.46 ; -1.95) | | | 0.1182 | -8.00 | (-14.15 ; -1.85) | | 0.1254 | |
|  | **Control** | -1.43 | (-8.14 ; 5.27) | | |  | -1.43 | (-8.11 ; 5.24) | |  |  |
| **Renal function monitoring** | **Exposure** | -4.73 | (-8.03 ; -1.42) | | | 0.01749 * | -4.12 | (-7.46 ; -0.79) | | 0.03744 * | |
|  | **Control** | 0.71 | (-2.83 ; 4.26) | | |  | 0.71 | (-2.91 ; 4.33) | |  |  |
| **Adequate eGFR levels** | **Exposure** | 3.79 | (-3.93 ; 11.52) | | | 0.7992 | 3.34 | (-4.26 ; 10.95) | | 0.8637 | |
|  | **Control** | 2.43 | (-5.85 ; 10.71) | | |  | 2.43 | (-5.83 10.69) | |  |  |

Table 2 - Estimates adjusted and unadjusted for hospital admission for the prolonged lockdown effect

|  | | **Post-lockdown trend** | | | | | | | | |
| --- | --- | --- | --- | --- | --- | --- | --- | --- | --- | --- |
|  |  | **Adjusted** | | | | **Unadjusted** | | | | |
|  |  | **Coefficient** | **Confidence interval** | | **P-value** | **Coefficient** | | **Confidence interval** | | **P-value** |
| **Lithium monitoring** | **Exposure** | 0.19 | (-0.00 ; 0.39) | 0.3796 | | 0.18 | (-0.02 ; 0.38) | | 0.4321 | |
|  | **Control** | 0.05 | (-0.20 ; 0.31) |  |  | 0.06 | (-0.20 ; 0.31) | |  |  |
| **Adequate Lithium levels** | **Exposure** | 0.63 | (-0.15 ; 1.41) | 0.2704 | | 0.73 | (-0.04 ; 1.50) | | 0.1983 | |
|  | **Control** | -0.05 | (-1.04 ; 0.95) |  |  | -0.06 | (-1.06 ; 0.94) | |  |  |
| **TSH monitoring** | **Exposure** | 0.14 | (0.01 ; 0.27) | 0.04411 * | | 0.13 | (-0.01 ; 0.26) | | 0.06064 . | |
|  | **Control** | -0.07 | (-0.24 ; 0.10) |  |  | -0.07 | (-0.24 ; 0.10) | |  |  |
| **Adequate TSH levels** | **Exposure** | 1.22 | (0.43 ; 2.00) | 0.6397 | | 1.19 | (0.42 ; 1.96) | | 0.6729 | |
|  | **Control** | 0.93 | (-0.07 ; 1.92) |  |  | 0.93 | (-0.06 ; 1.92) | |  |  |
| **Renal function monitoring** | **Exposure** | 0.34 | (-0.07 ; 0.76) | 0.2527 | | 0.26 | (-0.15 ; 0.68) | | 0.3989 | |
|  | **Control** | -0.03 | (-0.56 ; 0.50) |  |  | -0.02 | (-0.56 ; 0.52) | |  |  |
| **Adequate eGFR levels** | **Exposure** | -0.10 | (-1.07 ; 0.86) | 0.4773 | | -0.04 | (-0.99 ; 0.91) | | 0.5317 | |
|  | **Control** | 0.44 | (-0.79 ; 1.67) |  |  | 0.43 | (-0.80 ; 1.66) | |  |  |

.
